# The Extra Domain A of Fibronectin Increases VEGF-C Expression in Colorectal Carcinoma Involving the PI3K/AKT Signaling Pathway

**DOI:** 10.1371/journal.pone.0035378

**Published:** 2012-04-09

**Authors:** Lisha Xiang, Ganfeng Xie, Juanjuan Ou, Xing Wei, Feng Pan, Houjie Liang

**Affiliations:** Department of Oncology and Southwest Cancer Center, Southwest Hospital, Third Military Medical University, Chongqing, China; Virginia Commonwealth University, United States of America

## Abstract

The extra domain A (EDA)-containing fibronectin (EDA-FN), an alternatively spliced form of the extracellular matrix protein fibronectin, is predominantly expressed in various malignancies but not in normal tissues. In the present study, we investigated the potential pro-lymphangiogenesis effects of extra domain A (EDA)-mediated vascular endothelial growth factor-C (VEGF-C) secretion in colorectal carcinoma (CRC). We detected the expressions of EDA and VEGF-C in 52 human colorectal tumor tissues and their surrounding mucosae by immunohistochemical analysis, and further tested the correlation between the expressions of these two proteins in aforementioned CRC tissues. Both EDA and VEGF-C were abundantly expressed in the specimens of human CRC tissues. And VEGF-C was associated with increased expression of EDA in human CRC according to linear regression analysis. Besides, EDA expression was significantly correlated with lymph node metastasis, tumor differentiation and clinical stage by clinicopathological analysis of tissue microarrays containing tumor tissues of 115 CRC patients. Then, human CRC cell SW480 was transfected with lentivectors to elicit expression of shRNA against EDA (shRNA-EDA), and SW620 was transfected with a lentiviral vector to overexpress EDA (pGC-FU-EDA), respectively. We confirmed that VEGF-C was upregulated in EDA-overexpressed cells, and downregulated in shRNA-EDA cells. Moreover, a PI3K-dependent signaling pathway was found to be involved in EDA-mediated VEGF-C secretion. The *in vivo* result demonstrated that EDA could promote tumor growth and tumor-induced lymphangiogenesis in mouse xenograft models. Our findings provide evidence that EDA could play a role in tumor-induced lymphangiogenesis via upregulating autocrine secretion of VEGF-C in colorectal cancer, which is associated with the PI3K/Akt-dependent pathway.

## Introduction

Colorectal cancer (CRC) is the fourth most common malignancy worldwide with characteristic early metastasis. Lymphangiogenesis, associated with tumor metastasis, is evaluated in various tumor types, such as colon malignancies [1], esophageal carcinoma [2] and breast cancer [3]. Vascular endothelial growth factor (VEGF)-C is a most potent lymphangiogenic factor [4], which is correlated with lymph node metastasis in several tumors including CRC [5], [6]. Mechanically, the binding of VEGF-C to its receptor VEGFR-3 which is expressed on human lymphatic endothelial cells (LECs) can promote proliferation of lymphatic vessels [7], [8]. Thus, upregulation of VEGF-C production has been implicated in induction of tumor lymphangiogenesis and lymphatic invasion [9].

The understanding of the formation and the proliferation of new lymphatic vessels has been renewed by the discovery of tumor-induced lymphangiogenesis [10]. These concepts point out that tumors can express VEGF-C which upregulates VEGFR-3 expression of LECs and increases the number of lymphatic vessels in the vicinity of tumors [11]. Interestingly, lymphatic vessels surrounding VEGF-C-overexpressed tumors are multiplicated and grow intratumoraly from the border of tumors [12]. Many studies have reported that intratumoral lymphatics are present in several human tumors, which is sufficient to promote lymphatic metastasis [13].

It has been reported that VEGF-C is not only expressed in endothelial cells, but also expressed in non-endothelial cell types, including immune cells and cancer cells [14], [15]. Researchers have found that VEGF-C is overexpressed in various tumors including non-small-cell lung cancer (NSCLC), oral squamous cell cancer, undifferentiated gastric carcinoma, breast cancer, pancreatic cancer and colorectal carcinoma [15]. Although it is clear from many reports that overexpression of VEGF-C in a variety of human tumors correlates with tumor-induced lymphangiogenesis, it is less clear at what factors during tumor progression stimulate tumors to secret these lymphangiogenic factors.

Fibronectin (FN), which is an extracellular matrix cell-adhesive glycoprotein, contains three alternative splicing domains, extra domain A (EDA), extra domain B (EDB) and ΙΙΙCS [16], [17]. It has been reported that EDA is highly expressed in various malignancies but not in normal tissues [18], [19]. Our laboratory have previously observed that EDA could facilitate growth and tubulogenesis of LECs in the periphery of tumors [20], which indicated that EDA could contribute to tumor-associated lymphangiogenesis, but the underlying mechanisms remained to be defined. In this study, we found that upregulation of EDA in colorectal cancer cells could increase tumor cells autocrine secretion of VEGF-C both *in vitro* and *in vivo*, and then we explored the potential activation of intracellular signaling pathways. The results suggested that EDA could promote the secretion of VEGF-C in colorectal cancer cells, and this process was associated with the PI3K/Akt pathway.

## Results

### Expression and Correlation of EDA and VEGF-C in Human Colorectal Cancer Tissues

**Figure 1:**
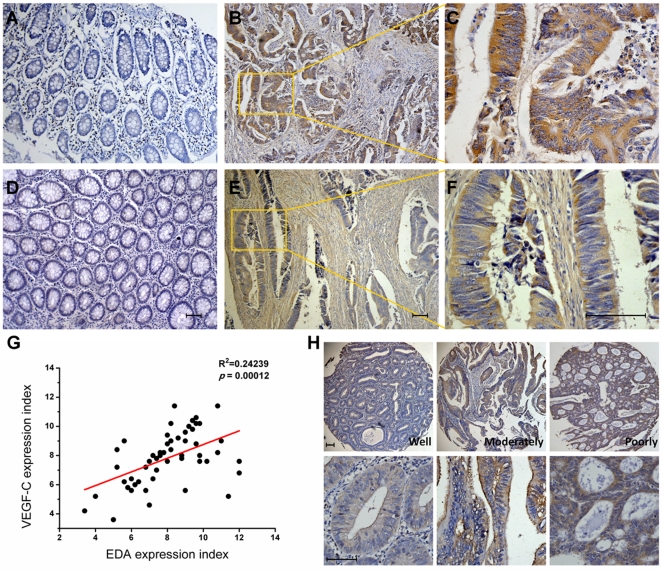
Immunohistochemical staining for EDA and VEGF-C in human colorectal carcinoma. Representative images of EDA expression in human colorectal carcinoma tissues (B) and in normal colorectal mucosae (A) are shown. Representative images of VEGF-C expression in colorectal carcinoma tissues (E) and in normal mucosae (D) are shown. Inset shows higher magnification of EDA(C) and VEGF-C (F) staining (brown), distinct from the colorectal benign mucosa. (G) Linear regression of EDA and VEGF-C of 52 human colorectal cancer samples was performed. (H) Representative images of EDA expression in tissue microarrays containing tumor samples from 115 CRC patients are shown. The bottom panel shows higher magnification of EDA staining. The slides were counterstained with hematoxylin. Scale bar = 50 µm.

To investigate the expression status of EDA and VEGF-C in colorectal cancer, we examined the expression of EDA and VEGF-C in human colorectal carcinoma samples and normal colorectal mucosae from 52 cases of CRC patients by immunohistochemical staining (IHC). The positive staining of EDA was indicated as yellow-brown precipitates in the cytoplasm in colorectal adenocarcinoma (Fig. 1B, 1C), but no positive staining has been seen in the adjacent normal non-cancerous colorectal tissues (Fig. 1A). Expression of VEGF-C in colorectal cancer tissues and cancer stroma was stained brown in the cytoplasm (Fig. 1E, 1F). In contrast, very little or no staining of VEGF-C was observed in normal mucosae (Fig. 1D). We further analyzed the correlation between EDA and VEGF-C expression in individual samples from 52 cases of CRC patients and found that EDA was significantly positively correlated with VEGF-C (*p*  =  0.00012) (Fig. 1G). Then, immunohistochemistry was performed to detect the expression of EDA protein in tissue microarrays containing tumor samples from 115 CRC patients. The immunostaining of EDA protein was substantially stronger in CRCs of clinically advanced stages (III and IV) or pathologically low grades (poorly and non-differentiated) relative to early stages (I and II) or high grades (well and moderately differentiated) (Fig. 1H). EDA was also highly expressed in tumor tissues of CRC patients with lymphatic metastasis compared with patients without lymphatic metastasis. The correlation of EDA expression with clinicopathological parameters of patients is shown in Table 1. High EDA expression was significantly correlated with present of lymph node invasion, tumor differentiation degree and advanced clinical stage (*p* < 0.05). The patient gender and age were not correlated with EDA expression (*p* > 0.05).

**Table 1:** Relationship between clinicopathological parameters of colon cancer cases and expression of EDA.

Variables	Total	Number of patients (%)		Chi-square test
		EDA low expression	EDA high expression	*P* value
**Age (year)**				
<61	49	15 (30.6)	34 (69.4)	0.092^a^
≥61	66	14 (21.2)	52 (78.8)	
**Gender**				
Male	64	19 (29.7)	45 (70.3)	0.078^a^
Female	51	10 (19.6)	41 (80.4)	
**Lymph node metastasis**				
Present	44	6 (13.6)	38 (86.4)	0.014^a^
Absent	71	23 (32.4)	48 (67.6)	
**Grade**				
well and moderately differentiated	85	25 (29.4)	60 (70.6)	0.041^b^
poorly and non-differentiated	30	4 (13.3)	26 (86.7)	
**Clinical stage**				
early stage	65	21 (32.3)	44 (67.7)	0.026^a^
advanced stage	50	8 (16.0)	42 (84.0)	

### Detection of Cellular and Secreted VEGF-C Protein in Transfected Cells and Control Cells

**Figure 2:**
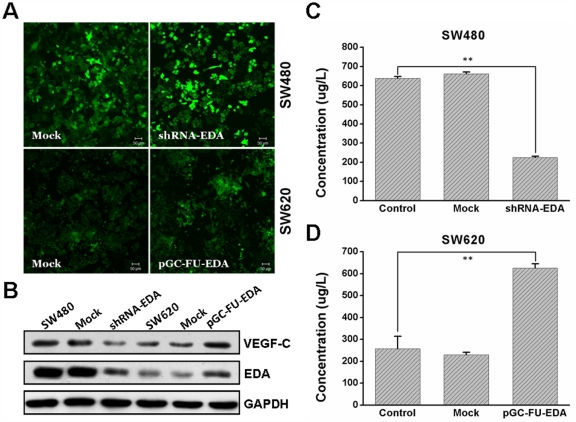
Expression of cellular and secreted VEGF-C protein in transfected cells and control cells. (A) shRNA-EDA SW480, mock SW480, pGC-FU-EDA SW620 and mock SW620 were confirmed by ﬂuorescent microscopy. (B) Expression of EDA and VEGF-C protein was determined by western blotting. GAPDH was a loading control. VEGF-C protein concentration in transfected SW480 group(C) and transfected SW620 group (D) was detected by ELISA. This experiment was repeated in duplicate more than three times. The error bars are the means ± SEM. and ** *p* < 0.01 is considered as statistically significant. Scale bar  = 50 µm.

In different types of human colorectal cancer cells, SW620 presents the lowest mRNA and protein level of EDA (data not shown), while SW480 expresses the highest (previously described in [20]). Thus, we generated pGC-FU-EDA cells (SW620 was transfected with a lentiviral vector to overexpress EDA) for comparison with nontransfected SW620 cells. SW480 was transfected with lentivectors to elicit expression of shRNA against EDA (shRNA-EDA). The transfection efficiency was observed to be approximately 70∼90% both in EDA-overexpressed cell group and shRNA-EDA cell group under the fluorescent microscopy (Fig. 2A). Then, we assessed the protein level of EDA and VEGF-C in transfected cells and control cells with Western blotting analysis (Fig. 2B). Compared with control counterparts, pGC-FU-EDA SW620 cells showed significantly increased expression levels of EDA and VEGF-C protein. In contrast, shRNA-EDA SW480 cells showed largely declined expression levels of EDA and VEGF-C protein.

ELISA test was performed to analyze the secretion of VEGF-C. The secretion of VEGF-C was largely increased in EDA-overexpressed cells supernatant compared with the control group (Fig. 2D, *p* < 0.01). Conversely, VEGF-C protein production was decreased in shRNA-EDA SW480 supernatant (Fig. 2C, *p* < 0.01). There was no obvious difference between the mock lentivector transfected tumor cells and nontransfected tumor cells.

### Effect of EDA on the PI3K/Akt Signaling Pathway of Colorectal Cancer Cells

**Figure 3:**
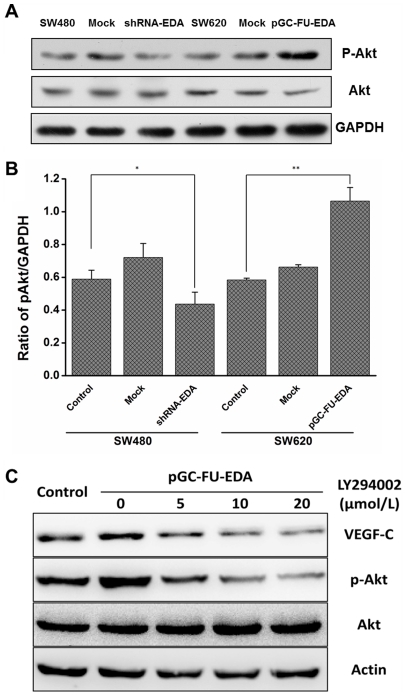
Activation of PI3K/Akt signaling pathway in transfected cells and control cells. (A) p-Akt and Akt proteins of SW620 group and SW480 group were performed by western blotting analysis. (B) Quantitative analysis shows the ratio of p-Akt/GAPDH. The error bars are standard deviations of three independent experimental results. * *p* < 0.05 and ** *p* < 0.01 are considered statistically significant and highly significant, respectively. (C) VEGF-C, p-Akt and Akt proteins of EDA-overexpressed SW620 cells were performed by western blotting when the EDA-overexpressed cells were preincubated in medium containing 0–20 µM LY294002 for 24 h. Wild type SW620 was set up as the control group. The results shown are the average of three experiments.

PI3K/Akt pathway activation is known to mediate signal transduction of several growth factors. It has been reported that type I insulin-like growth factor receptor (IGF-IR) induces VEGF-C expression in an Akt-dependent pathway [21]. Thus, to investigate how EDA regulates VEGF-C, we checked the expression of phosphorylated Akt in the transfected group and the control group. Western blotting analysis showed that the increased level of phosphorylated Akt was detected in pGC-FU-EDA SW620 cells, while the expression of p-Akt in shRNA-EDA SW480 cells was decreased significantly. There was no significant difference between mock lentivector transfected tumor cells and nontransfected tumor cells (Fig. 3A, 3B). To identify the PI3K/Akt signaling pathway involved in EDA-mediated induction of VEGF-C, we examined the effect of PI3-Kinase specific inhibitor (LY294002). Dose-dependent reductions of VEGF-C expression were observed when the EDA-overexpressed cells were cultured with 0 µM, 5 µM, 10 µM, or 20 µM LY294002 in the absence of FBS for 24 h (Fig. 3C). LY294002 (0–20 µM) significantly reduced the levels of phosphorylated Akt in EDA-overexpressed cells in a concentration-dependent manner, but the levels of total Akt were not changed (Fig. 3C). PI3K/Akt signaling pathway activation thus might play a role in the EDA-mediated VEGF-C secretion.

### The Tumorigenicity and Expression of EDA and VEGF-C in Nude Mouse Xenograft Models of Colorectal Carcinoma

**Figure 4:**
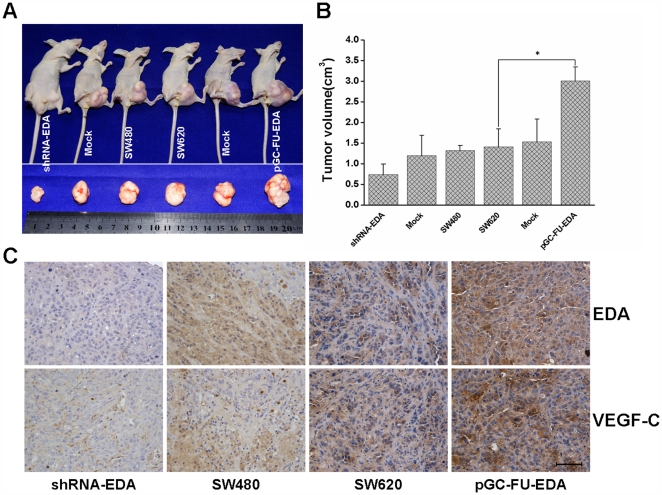
BALB/c nude mice were subcutaneouly injected with transfected cells and negative control cells. Xenografts were excised and sized 42 days later. (A) Effect of EDA on tumor proliferation. (B) Tumor volumes were measured when mice were sacrificed and the data are presented as mean determinants (±SEM). * *p* < 0.05. (C) Immunohistochemical staining of EDA and VEGF-C was performed in nude mouse xenografts. Scale bar  = 50 µm.

We established nude mouse xenograft models in which pGC-FU-EDA SW620 cells, shRNA-EDA SW480 cells and control cells were subcutaneously injected in the left inguina. The solid tumors became directly visible by gross examination 2 weeks after implantation. Tumor sizes were detected by measuring with vernier caliper after 6 weeks of tumor growth (data shown in Fig. 4B, *p* < 0.05). Autopsy analysis showed that the xenografts derived form pGC-FU-EDA SW620 cells were grown bigger than those developed from SW620 cells or mock group as measured by tumor weight and volume (Fig. 4A). In contrast, the subcutaneous tumors developed from shRNA-EDA SW480 cells were grown distinctly smaller than those in the control group (Fig. 4A).

Immunohistochemical staining revealed that the staining intensity of EDA and VEGF-C in pGC-FU-EDA SW620 tumor group was enhanced in comparison with that in nontransfected control group or mock lentivector transfected group, and the staining intensity in shRNA-EDA SW480 tumor group was extremely diminished in comparison with that in the control group (Fig. 4C).

### The Tumorigenicity of Human CRC in an Orthotopic Nude Mouse Model and Effect of EDA on Intratumoral Lymphangiogenesis *in Vivo*

**Figure 5:**
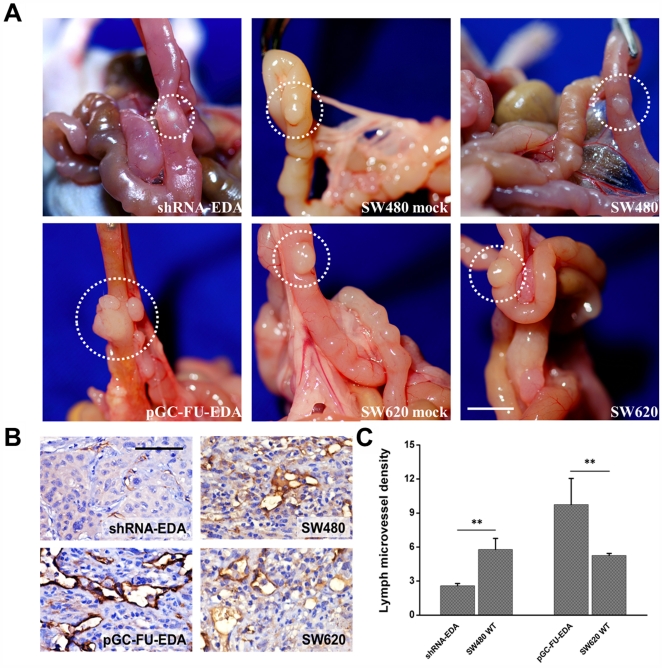
Orthotopic xenografts derived from transfected cells and control cells. Seven to 8 wk old male BALB/c nude mice were orthotopically implanted with 6 groups of tumor xenografts. (A) Representative images of xenografts. Orthotopic images of xenograft tumors derived from 6 groups of transfected cells and control cells. Scale bar = 5 mm. (B) Expression of LYVE-1 was used to count LMVD in xenografts by immunohistochemical staining. The distribution of lymph vessels was stained brown under the light microscope. Scale bar  = 50 µm. (C) Quantification of lymph microvessel density (LMVD) is shown. The results shown are the average of three experiments. ** p < 0.01, as compared with control group. The values are displayed as the mean ± SEM.

pGC-FU-EDA SW620 cells, shRNA-EDA SW480 cells and control cells were implanted orthotopically into nude mice to analyze their tumorigenic potential. All cell lines formed tumors 8 weeks after implantation. Tumors formed by pGC-FU-EDA SW620 cells grew most rapidly compared with those formed by nontransfected control cells or mock lentivector transfected cells, while tumors formed by shRNA-EDA SW480 cells were the smallest and grew more slowly than those control group cells (Fig. 5A).

To investigate whether EDA upregulating the secretion of VEGF-C can contribute to intratumoral lymphangiogenesis *in vivo*, we examined the number of intratumoral lymphatic vessels by immunohistochemistry analysis. The results showed that there was a higher LMVD in pGC-FU-EDA SW620 tumor group as compared with that in control group (Fig. 5B). However, an extremely small number of intratumoral lymphatic vessels were found in shRNA-EDA SW480 tumor group (Fig. 5B). Right panel shows the quantification data about the lymph microvessel density (LMVD) (Fig. 5C, *p* < 0.01).

## Discussion

Lymphangiogenesis or the growth of lymphatic vessels is an important step in tumor metastasis. Lymphangiogenesis and early regional metastasis frequently occurs in several kinds of malignant tumors including colorectal cancer. Metastasis to regional lymph nodes is generally believed as the first indication that a tumor has progressed to metastatic competence. However, many studies have cast new light on the biology of lymphangiogenesis and molecular mechanisms of tumor regional lymph nodes metastasis. One of the mechanisms is tumor-induced lymphangiogenesis. Evidences of intratumoral lymphatic vessels raise the possibility that tumor cells can contribute to lymphatic metastasis through the induction of a lymphangiogenic process [22]. It is well known that tumor cells enter the lymphatic vasculature by eliciting lymphangiogenesis via growth factor production. Moreover, lymphangiogenic growth factors produced by tumor cells stimulate growth and dilation of the tumor-induced lymphatic vessels, as well as facilitating tumor regional lymph nodes metastasis. VEGF-C, as one of the lymphangiogenic growth factors, is a key regulator in lymphangiogenesis and tumor metastasis [23]. Early studies suggested that VEGF-C could promote the growth of new lymphatic vessels and regional metastasis by binding to their receptor tyrosine kinase VEGFR-3 which was expressed abundantly in lymphatic endothelial cells [24], [25]. Studies with human or animal tumor models implicated that malignant tumor cells themselves could secrete high levels of VEGF-C [26], and this overexpression of tumor-derived VEGF-C might play an important role in intratumorally-occurred lymphangiogenesis, which would in turn promote dissemination of tumor cells to regional lymph nodes [27]–[29].

The stimulation by many growth factors, including IGF-1, PDGF, EGF, and TGF-β, has been shown to induce the expression of VEGF-C in malignant tumors [30]. In our previous study, we investigated the fibronectin alternatively spliced EDA domain and its effects on lymphatic neovasculature of colorectal carcinoma [20]. We found that EDA promoted cytoskeleton organization and tubulogenesis capability of LECs. These results suggested that EDA could be regarded as a promising factor of tumor-associated lymphangiogenesis. But a systematic study of this factor on the molecular mechanisms of lymphangiogenesis has not been conducted. In this study, we wanted to investigate the possible regulation mechanisms of EDA to the growth of lymphatic vessels.

According to our work, we hypothesized that EDA stimulates lymphangiogenesis through the following two potential mechanisms: (1) direct stimulation of LECs growth; and (2) stimulation of autocrine secretion of VEGF-C by tumor cells themselves. As for the first mechanism, combined with the fact that integrin α9β1 is a structurally related receptor of EDA [31] and our previous study in which we found that integrin α9 was upregulated when LECs were treated with the exogenous EDA, a conclusion could be drawn that EDA-induced lymphangiogenesis might partly depend on the activation of integrin α9 of LECs. We presumed the other potential mechanism is that EDA may stimulate tumor-induced lymphangiogenesis via upregulating autocrine secretion of VEGF-C in colorectal cancer cells. It is known that VEGF-C can promote the proliferation of LECs. In addition, we found that elevated expression of EDA significantly correlated with the overexpression of VEGF-C in colorectal cancer tissues by immunohistochemistry analysis. To investigate the possibility of this mechanism, we have generated EDA-overexpressed tumor cells (pGC-FU-EDA) and shRNA-EDA tumor cells. Western blotting analysis exhibited that EDA-overexpressed cell group was detected increased level of EDA and VEGF-C protein. Conversely, shRNA-EDA cell group expressed decreased level of EDA and VEGF-C. By virtue of these, we tested the secreted VEGF-C protein in transfected cells and control cells by ELISA. The EDA-overexpressed SW620 group had a significantly upregulated VEGF-C protein level compared with the nontransfected SW620 group. By contrast, the level of secreted VEGF-C was largely depressed in supernatant of shRNA-EDA SW480 cells. All of these results implicated that EDA could contribute to the excretion of VEGF-C in colorectal cancer.

To corroborate these findings further, we established nude mouse xenograft models. Tumors formed by shRNA-EDA SW480 cells were the smallest and grew more slowly, while tumors formed by EDA-overexpressed SW620 cells grew more rapidly after implantation. Moreover, immunohistochemical staining showed that both the staining intensity of VEGF-C and the number of intratumoral lymphatic vessels in EDA-overexpressed SW620 tumor group were increased, while both of them were reduced in shRNA-EDA SW480 tumor group. These results suggested that EDA could facilitate tumor proliferation and VEGF-C-mediated tumorous lymphangiogenesis. By virtue of these, we verified these results by analyzing the relationship between EDA and clinicopathological parameters of CRC patients. We detected the expression of EDA protein in tissue microarrays containing tumor samples from 115 CRC patients and found that EDA expression was significantly correlated with present of lymph node invasion, tumor differentiation degree and advanced clinical stage.

Furthermore, we studied the signaling pathway involved in EDA-mediated tumor-derived VEGF-C secretion. The expressional regulation of VEGF family has been well investigated. For instance, hypoxia induces VEGF-A expression in an PI3K/Akt-dependent pathway [32]. And PI3K/Akt signaling pathway is also involved in IGF-1-induced VEGF-C expression in lung carcinoma cells [21]. It is well known that PI3K can mediate the phosphorylation and activation of its downstream serine/threonine kinase, Akt (or PKB), participating in some important biological activities such as survival, proliferation, migration and differentiation in human cancer [33].The activation of Akt also contributes to tumorigenesis and tumor metastasis in various types of human cancer [34], [35]. By exploring well-characterized pathways known to regulate tumor-derived VEGF-C expression, we found that PI3K/Akt signal transduction pathway probably plays a pivotal role in the EDA-mediated regulation of VEGF-C expression in human colorectal carcinomas. We performed Western blot to study the activation of PI3K/Akt signaling pathway in transfected cells and control cells. In EDA-overexpressed cells, the levels of phospho-Akt were more activated than nontransfected cells in response to EDA. Additionally, we found that cells pretreated with PI3K specific inhibitor LY294002 could inhibit EDA-induced Akt phosphorylation, and consequently caused a significant inhibition of VEGF-C induction. Impressively, the blockage of these intracellular signaling components and VEGF-C induction was concentration-dependent. These results indicate that the PI3K/Akt pathway is involved in the expressional regulation of EDA-mediated VEGF-C in colorectal cancer cells.

In conclusion, our findings support the hypothesis that during the process of tumor-induced lymphangiogenesis, the extra domain A of fibronectin could promote the creation of new lymphatic vessels and one of its mechanisms may be that EDA could enhance VEGF-C concentration in colorectal cancer, and the activation of the PI3K/Akt signaling pathway is involved in this upregulation. Considering all of these, EDA and its effects on activating intracellular signaling pathways may open novel imaging opportunities and targeted therapeutic modalities in dealing with lymphatic metastasis in colorectal carcinoma.

## Materials and Methods

### Ethics Statement

Experiments using the animals were conducted with the approval of the Animal Care and Use Committee of Third Military Medical University (Approval ID: SCXK (Military) 2007015), according to the State Science and Technology Commission Regulations for the Administration of Affairs Concerning Experimental Animals (1988, China).

The clinical investigation was complied with the Helsinki Declaration. The protocol of immunohistochemistry for patient tissues was approved by the Ethics Committee of Southwest Hospital, Third Military Medical University (Permit Number: 2009[21]), and all patients provided written consent form.

### Cell Lines and Culture Conditions

The human colorectal carcinoma cell lines SW620, SW480 and HEK293T were obtained from the American Type Culture Collection (Manassas, VA, USA). Cell culture reagents were purchased from Gibco-Invitrogen (Carlsbad, CA, USA). Cells were maintained in Dulbecco’s Modified Eagle’s Medium, supplemented with 10% fetal bovine serum, 100 U/ml penicillin and 0.1 mg/ml streptomycin at 37°C in an atmosphere containing 5% CO_2_.

### Construction and Preparation of Lentivirus for RNAi and Overexpression of EDA

shRNA-EDA (GGATGGAATCCATGAGCTA) was designed against EDA (accession number: NM212482) and synthesized as follows: Forward: 5′- CcgggaGGATGGAATCCATGAGCTATTCAAGAGATAGCTCATGGATTCCATCCtcTTTTTg-3′, and Reverse: 5′- aattcaaaaagaGGATGGAATCCATGAGCTATCTCTTGAATAGCTCATGGATTCCATCCtc-3′. These oligos were annealed and inserted downstream of the U6 promoter on the lentiviral vector pGCL-GFP (GeneChem). A control vector containing non-silencing sequence (5′-TTCTCCGAACGTGTCACGT-3′) was supplied by GeneChem Co., Ltd. (Shanghai, China). For overexpression of EDA, ORF of EDA was cloned into the lentiviral vector expression plasmid pGC-FU (GeneChem).

Lentiviruses were generated by triple transfection of 80% confluent HEK293T cells with pGCL-GFP-shEDA or pGC-FU-EDA plasmid, together with pHelper 1.0 and pHelper 2.0 helper plasmids (GeneChem) using Lipofectamine 2000 (Invitrogen, Carlsbad, CA, USA).

Lentivirus was harvested at 48h and 72 h post transfection, centrifuged to remove cell debris, and then filtered through a 0.45 µm cellulose acetate filter followed by ultracentrifugation. For lentivirus infection, SW480 and SW620 cells were grown to 70–80% confluence and infected with pGCL-GFP-shEDA lentivirus, pGC-FU-EDA lentivirus or control lentivirus, separately, at MOI of 10 (SW480) or 50 (SW620). To determine the infection efficiency, cells expressing GFP protein were imaged using laser confocal scanning microscopy (Leica TSC-SP5, Germany) 4 days after infection. The GFP positive cells were purified with a FACSCalibur flow cytometer (BD Biosciences).

### Inhibition of Signal Transduction Kinase

EDA-overexpressed SW620 cells were seeded at a density of 1.0×10^5^/ml cells/well in 6-well plates and incubated in serum-free medium overnight. LY294002 (Sigma Chemical Co., St. Louis, MO) was dissolved in DMSO at a stock concentration of 10 mM and added to cell cultures at a concentration of 0 µM, 5 µM, 10 µM, or 20 µM. The final concentration of DMSO used in our study did not affect cell survival or protein phosphorylation. Cells were treated by LY294002 for 24 h and then prepared for Western blot.

### Tissue Microarrays and Immunohistochemistry (IHC)

One hundred and fifteen colorectal cancers were made into tissue microarrays using the tissuearrayerTMA-1(Beecher Instruments, WA, USA) as described previously[36]. Fifty-two samples including colorectal cancers and their surrounding tissues as well as xenografts were collected and processed into formalin-fixed paraffin-embedded tissue blocks, then cut into 4 µm-thickness sections. The tissue microarrays and tumor sections were routinely dewaxed in xylene, rinsed in graded ethanol, and finally rehydrated in double-distilled water. Endogenous peroxidase activity was blocked by incubation in 3% hydrogen peroxidemethanol for 15min. Antigen retrieval was accomplished by heating the slides in 1 mM EDTA solution (pH 8.0). After washing in phosphate-buffered saline and exposure to 10% normal goat serum for 10 min to reduce nonspecific binding, the slides were incubated overnight at 4°C with a 1∶100 dilution of goat anti-human VEGF-C polyclonal antibody (Abcam, Cambridge, MA, USA) or a 1∶100 dilution of mouse anti-human EDA polyclonal antibody (Santa Cruz Biotechnology, CA, USA). The tissue microarrays and sections were stained using the SP method according to the kit instructions. The instantaneous SP supersensitive kits (PV-9003 and PV-6002) were provided by Beijing Zhongshan Jinqiao biotechnology Co., Ltd.

### Criteria for Assessing Immunohistochemical Results

For each slide, five random fields were selected for scoring and a mean score of each slide was calculated in final analysis. Positive staining was accessed using a five scoring system: 0 (no positive cells), 1 (<10% positive cells), 2 (10%–40% positive cells), 3 (40%–70% positive cells), and 4 (>70% positive cells). To achieve accuracy, the intensity of positive staining was also used in a four scoring system: 0 (negative staining), 1 (weak staining exhibited as light yellow), 2 (moderate staining exhibited as yellow brown), and 3 (strong staining exhibited as brown). Protein expression index  =  (intensity score) × (positive score). To test the correlation between EDA and VEGF-C, linear regression analysis was performed based on expression index of each protein.

### ELISA Assay

Cells were put into 25 ml culture bottles at the density of 2.0×10^5^/ml. After 24h in culture, supernatants of these cells were harvested in sterile conditions respectively, centrifuged to remove eventual dead cells. Supernatants were centrifuged at 1,200 rpm for 5 min at 4°C. The levels of VEGF-C secretion were measured using mouse enzyme-linked immunosorbent assay (ELISA) kits (Shanghai Hushang Biotechnology Co., Ltd.). According to the instructions of ELISA reagent kit, the light absorption value at 450 nm wavelength was measured with microplate reader (Bio-Rad, Hercules, CA). The experiment was repeated three times and each time we would use three parallel wells.

### Western Blotting Analysis

Cell extracts were prepared and Western blotting were performed according to the instruction of RIPA buffer (Biotek Corporation, Beijing, China). Cell lysates were collected by centrifugation at 12,000 rpm for 15 min at 4°C, and then transferred to clean microcentrifuge tubes. Protein concentration was determined with Bradford reagent (Bio-Rad), and equal amounts of proteins (50 µg) were run on a 10% SDS–PAGE gel and blotted onto polyvinylidene fluoride membranes. After blocking for 2h at room temperature with 5% non-fat-dry milk, membranes were incubated with anti-VEGF-C goat polyclonal antibody, anti-EDA mouse polyclonal antibody, Akt antibody (Cell Signaling Technology; 1/2000), phospho-Akt antibody (Cell Signaling Technology;1/2000) and anti-GAPDH antibodies (Santa Cruz Biotechnology) at 4°C overnight, respectively. The secondary antibody was HRP-conjugated anti-IgG (Boster Biotechnology, Wuhan, China). Membranes were then incubated with SuperSignal West Femto Maximum Sensitivity Substrate (Pierce, Rockford, IL, USA) for 1minute and imaged using a Gel Doc XR system (Bio-Rad). Antibodies were removed with stripping buffer (Pierce) at 50°C for 30 min, followed by washing with PBS Tween 20, and membranes were reprobed.

### Animals and Subcutaneous Implantation of Tumors

Male athymic BALB/c nude mice (4–5 weeks old) were purchased from the Institute of Experimental Animal of Third Military Medical University (Chongqing, China). Mice were maintained under specific pathogen-free conditions. All animals had free access to standard laboratory mouse food and water. Cells (1.0×10^6^) in 0.1 ml PBS were injected subcutaneously into the groin of male athymic BALB/c nude mice respectively. Mice were randomly chosen and assigned to 6 groups (3 mice per group) based on the difference of cells:SW620 control group, pGC-FU-EDA SW620 group, Mock-SW620 group,SW480 control group, shRNA-EDA SW480 group, Mock-SW480 group. Tumor growth was monitored at a defined regular interval (5 days) by measuring diameters using vernier caliper. Tumor volume was determined based on the following formula: volume = 0.52 ab^2^, where a = long diameter and b = short diameter. After a 42-day follow-up period, mice were sacrificed and the tumors were removed. The tumors were fixed in 10% buffered formalin for IHC.

### Animals and Orthotopic Implantation of Tumors

Male athymic BALB/c nude mice (7–8 weeks old) were purchased from the Institute of Experimental Animal of Third Military Medical University (Chongqing, China). Mice were maintained under specific pathogen-free conditions. All animals had free access to standard laboratory mouse food and water. To establish orthotopic xenografts, cells (1.0×10^7^) in 200 µl PBS were injected into the colon subserosa of male athymic BALB/c nude mice respectively. Mice were randomly chosen and assigned to 6 groups (5 mice per group) based on the difference of cells: SW620 control group, pGC-FU-EDA SW620 group, Mock-SW620 group, SW480 control group, shRNA-EDA SW480 group, Mock-SW480 group. Stool shape and abdominal pertrusion in the animals were observed every other day. Subsequently, five mice of each group were sacrificed 8 weeks after the cancer cell injection. Xenografts were harvested for section preparation. Tumor volume was determined based on the following formula: volume = 0.52 ab^2^, where a = long diameter and b = short diameter.

### Immunohistochemistry Analysis and Lymph Microvessel Density Quantification

For immunohistochemical staining of orthotopic xenografts, tumor tissue specimens were fixed in 10% buffered formalin. The fixed tissues were embedded in paraffin, and serial sections were made and stained with a 1∶200 dilution of a rabbit polyclonal antibody against LYVE-1 (Abcam, Cambridge, MA, USA) for lymph microvessel density analysis. The sections were stained using the SP method according to the kit instructions. Under the light microscope, lymph microvessel density was determined by counting the number of lymph microvessels per 3 high power fields (x100) in the sections.

### Statistical Analysis

Statistical analysis of the *in vitro* and *in vivo* results was analyzed using SPSS 13.0 software (Version 13.0, LEAD Technologies, Chicago, USA). To compare the statistical significance of differential findings among different experimental groups, one-way analysis of variance (ANOVA) was performed. The quantitative data were expressed as the mean ± SEM of three to five independent experiments. *p* values below 0.05 (*) and 0.01 (**) were considered statistically significant and highly significant, respectively.

## References

[1] Sundlisaeter E, Dicko A, Sakariassen PØ, Sondenaa K, Enger PØ (2007). Lymphangiogenesis in colorectal cancer--prognostic and therapeutic aspects.. Int J Cancer.

[2] Saad RS, Lindner JL, Liu Y, Silverman JF (2009). Lymphatic vessel density as prognostic marker in esophageal adenocarcinoma.. Am J Clin Pathol.

[3] Liu HT, Ma R, Yang QF, Du G, Zhang CJ (2009). Lymphangiogenic characteristics of triple negativity in node-negative breast cancer.. Int J Surg Pathol.

[4] Clasper S, Royston D, Baban D, Cao Y, Ewers S (2008). A novel gene expression profile in lymphatics associated with tumor growth and nodal metastasis.. Cancer Res.

[5] Barresi V, Reggiani-Bonetti L, Di Gregorio C, De Leon MP, Barresi G (2011). Lymphatic vessel density and its prognostic value in stage I colorectal carcinoma.. J Clin Pathol.

[6] Duff SE, Li C, Renehan A, O’Dwyer ST, Kumar S (2003). Immunodetection and molecular forms of plasma vascular endothelial growth factor-C.. Int J Oncol.

[7] Kim JG, Chae YS, Sohn SK, Cho YY, Moon JH (2008). Vascular endothelial growth factor gene polymorphisms associated with prognosis for patients with colorectal cancer.. Clin Cancer Res.

[8] Parr C, Jiang WG (2003). Quantitative analysis of lymphangiogenic markers in human colorectal cancer.. Int J Oncol.

[9] Royston D, Jackson DG (2009). Mechanisms of lymphatic metastasis in human colorectal adenocarcinoma.. J Pathol.

[10] Krishnan J, Kirkin V, Steffen A, Hegen M, Weih D (2003). Differential in vivo and in vitro expression of vascular endothelial growth factor (VEGF)-C and VEGF-D in tumors and its relationship to lymphatic metastasis in immunocompetent rats.. Cancer Res.

[11] Karpanen T, Egeblad M, Karkkainen MJ, Kubo H, Ylä-Herttuala S (2001). Vascular endothelial growth factor C promotes tumor lymphangiogenesis and intralymphatic tumor growth.. Cancer Res.

[12] Beasley NJ, Prevo R, Banerji S, Leek RD, Moore J (2002). Intratumoral lymphangiogenesis and lymph node metastasis in head and neck cancer.. Cancer Res.

[13] Mandriota SJ, Jussila L, Jeltsch M, Compagni A, Baetens D (2001). Vascular endothelial growth factor-C-mediated lymphangiogenesis promotes tumour metastasis.. EMBO J.

[14] Achen MG, McColl BK, Stacker SA (2005). Focus on lymphangiogenesis in tumor metastasis.. Cancer Cell.

[15] Pepper MS (2001). Lymphangiogenesis and tumor metastasis: myth or reality?.

[16] Villa A, Trachsel E, Kaspar M, Schliemann C, Sommavilla R (2008). A high-affinity human monoclonal antibody specific to the alternatively spliced EDA domain of fibronectin efficiently targets tumor neo-vasculature in vivo.. Int J Cancer.

[17] Romberger DJ (1997). Fibronectin.. Int J Biochem Cell Biol.

[18] McFadden JP, Baker BS, Powles AV, Fry L (2010). Psoriasis and extra domain A fibronectin loops.. Br J Dermatol.

[19] van der Straaten HM, Canninga-van Dijk MR, Verdonck LF, Castigliego D, Borst HP (2004). Extra-domain-A fibronectin: a new marker of fibrosis in cutaneous graft-versus-host disease.. J Invest Dermatol.

[20] Ou JJ, Wu F, Liang HJ (2010). Colorectal tumor derived fibronectin alternatively spliced EDA domain exserts lymphangiogenic effect on human lymphatic endothelial cells.. Cancer Biology & Therapy.

[21] Tang Y, Zhang D, Fallavollita L, Brodt P (2003). Vascular endothelial growth factor C expression and lymph node metastasis are regulated by the type I insulin-like growth factor receptor.. Cancer Res.

[22] Oliver G, Detmar M (2002). The rediscovery of the lymphatic system: old and new insights into the development and biological function of the lymphatic vasculature.. Genes Dev.

[23] Caunt M, Mak J, Liang WC, Stawicki S, Pan Q (2008). Blocking neuropilin-2 function inhibits tumor cell metastasis.. Cancer Cell.

[24] Bahram F, Claesson-Welsh L (2010). VEGF-mediated signal transduction in lymphatic endothelial cells.. Pathophysiology.

[25] Girling JE, Rogers PA (2009). Regulation of endometrial vascular remodelling: role of the vascular endothelial growth factor family and the angiopoietin-TIE signalling system.. Reproduction.

[26] Saintigny P, Kambouchner M, Ly M, Gomes N, Sainte-Catherine O (2007). Vascular endothelial growth factor-C and its receptor VEGFR-3 in non-small-cell lung cancer: concurrent expression in cancer cells from primary tumour and metastatic lymph node.. Lung Cancer.

[27] Chang L, Kaipainen A, Folkman J (2002). Lymphangiogenesis new mechanisms.. Ann N Y Acad Sci.

[28] Su JL, Chen PS, Chien MH, Chen PB, Chen YH (2008). Further evidence for expression and function of the VEGF-C/VEGFR-3 axis in cancer cells.. Cancer Cell.

[29] Tammela T, Alitalo K (2010). Lymphangiogenesis: Molecular Mechanisms and Future Promise.. Cell.

[30] Enholm B, Paavonen K, Ristimäki A, Kumar V, Gunji Y (1997). Comparison of VEGF, VEGF-B, VEGF-C and Ang-1 mRNA regulation by serum, growth factors, oncoproteins and hypoxia.. Oncogene.

[31] Avraamides CJ, Garmy-Susini B, Varner JA (2008). Integrins in angiogenesis and lymphangiogenesis.. Nature Reviews Cancer.

[32] Skinner HD, Zheng JZ, Fang J, Agani F, Jiang BH (2004). Vascular endothelial growth factor transcriptional activation is mediated by hypoxia-inducible factor 1alpha, HDM2, and p70S6K1 in response to phosphatidylinositol 3-kinase/AKT signaling.. J Biol Chem.

[33] Saji M, Ringel MD (2010). The PI3K-Akt-mTOR pathway in initiation and progression of thyroid tumors.. Mol Cell Endocrinol.

[34] West KA, Castillo SS, Dennis PA (2002). Activation of the PI3K/Akt pathway and chemotherapeutic resistance.. Drug Resist Updat.

[35] Goc A, Al-Husein B, Kochuparambil ST, Liu J, Heston WW (2011). PI3 kinase integrates Akt and MAP kinase signaling pathways in the regulation of prostate cancer.. International Journal of Oncology.

[36] Duan GJ, Yan XC, Bian XW, Li J, Chen X (2004). [The significance of beta-catenin and matrix metalloproteinase-7 expression in colorectal adenoma and carcinoma].. Zhonghua bing li xue za zhi Chinese journal of pathology.

